# Population structure of Environmental and Clinical *Legionella pneumophila* isolates in Catalonia

**DOI:** 10.1038/s41598-018-24708-1

**Published:** 2018-04-19

**Authors:** Sara Quero, Noemí Párraga-Niño, Irene Barrabeig, Maria Rosa Sala, M. Luisa Pedro-Botet, Eduard Monsó, Mireia Jané, Miquel Sabrià, Marian Garcia-Núñez

**Affiliations:** 1 Unitat de Malalties Infeccioses, Fundació Institut d’Investigació Germans Trias I Pujol, Hospital Universitari Germans Trias i Pujol, Badalona, Spain; 2 CIBER de Enfermedades Respiratorias, CIBERES, Madrid, Spain; 3 Department of Medicine, Universitat Autònoma de Barcelona (UAB), Barcelona, Spain; 4 Department of Respiratory Medicine, Parc Taulí Hospital Universitari. Institut d’Investigació i Innovació Parc Taulí, I3PT Sabadell, Spain; 5 Vigilancia Epidemiologica, Agencia de Salut Pública de Catalunya, Barcelona, Spain

## Abstract

*Legionella* is the causative agent of Legionnaires’ disease (LD). In Spain, Catalonia is the region with the highest incidence of LD cases. The characterisation of clinical and environmental isolates using molecular epidemiology techniques provides epidemiological data for a specific geographic region and makes it possible to carry out phylogenetic and population-based analyses. The aim of this study was to describe and compare environmental and clinical isolates of *Legionella pneumophila* in Catalonia using sequence-based typing and monoclonal antibody subgrouping. A total of 528 isolates were characterised. For data analysis, the isolates were filtered to reduce redundancies, and 266 isolates (109 clinical and 157 environmental) were finally included. Thirty-two per cent of the clinical isolates were ST23, ST37 and ST1 while 40% of the environmental isolates were ST284 and ST1. Although the index of diversity was higher in clinical than in environmental ST isolates, we observed that clinical STs were similar to those recorded in other regions but that environmental STs were more confined to particular study areas. This observation supports the idea that only certain STs trigger cases or outbreaks in humans. Therefore, comparison of the genomes of clinical and environmental isolates could provide important information about the traits that favour infection or environmental persistence.

## Introduction

*Legionella* spp. is the causative agent of Legionnaires’ disease (LD), and *Legionella pneumophila* is associated with the majority of LD cases^1–4^. The main reservoirs of this bacterium are water-related natural habitats, where it forms biofilms in surface interphases or survives within amoeba. *Legionella* can colonise a range of man-made water distribution systems, in which it multiplies actively. These sources have been associated with LD cases and outbreaks^5–9^.

Notification of LD cases to the health authorities has been mandatory since 1988 in Catalonia and since 1996 in Spain. In Catalonia the median burden of LD was 3.7 per 100 000 inhabitants (2008–2016) and it is the Spanish region with the highest number of reported cases. Despite this high incidence, environmental *L*. *pneumophila* populations in Catalonia have not been systematically characterised and only one report has described clinical isolates of this bacterium^9^.

Sequence-based typing (SBT) is a simple, fast, discriminatory and reproducible molecular typing method for characterising the distribution of *L*. *pneumophila* isolates^10–12^. The characterisation of clinical and environmental isolates using molecular epidemiology techniques provides data for a specific geographic region and makes it possible to perform phylogenetic and population-based analyses. This characterisation is essential for a better understanding of the transmission routes of environment-associated bacteria. The results of the distribution of sequence types (STs) of *L*. *pneumophila* isolates from Europe, Asia and North America showed a predominance of specific groups of STs in clinical isolates, and suggest that most clinical cases and outbreaks are caused by a reduced number of *L*. *pneumophila* isolates^13–20^.

The aim of this study was to describe and compare clinical and non-related environmental *L*. *pneumophila* isolates in Catalonia using sequence-based typing and monoclonal antibody (MAb) subgrouping.

## Results

### *L*. *pneumophila* identification

Between 1989 and 2016, 528 isolates were included in the study and were characterised by MAbs and SBT. After eliminating related isolates and environmental replicates from the same points of analysis, the comparison was performed with 266 isolates (109 clinical and 157 environmental).

Only 13.5% (36/266) of these isolates were *L*. *pneumophila* non-sg 1. A higher proportion of *L*. *pneumophila* non-sg 1 isolates was observed in the environmental group (clinical: 8.26% vs. environmental: 17.20%; p = 0.002). All community-acquired (CA) clinical isolates were identified as *L*. *pneumophila* sg 1, while 27.3% of hospital-acquired (HA) clinical isolates were *L*. *pneumophila* non-sg 1 (p < 0.0001).

### Reactivity against MAb 3/1

**Figure 1:**
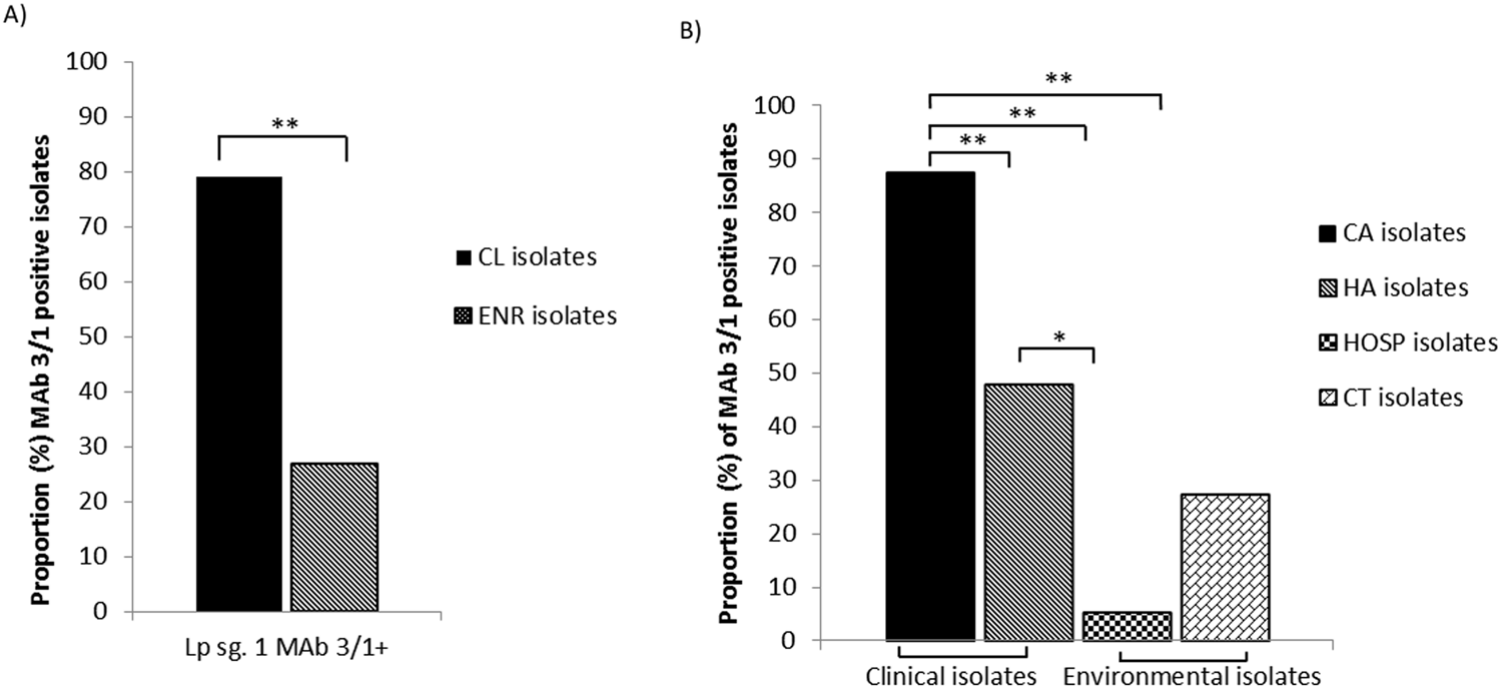
Distribution of the MAb3/1 positive isolates according to acquisition. Statistical differences between groups are shown as *(p < 0.01) and **(p < 0.005). (**A**) CL: clinical isolates; ENR: environmental isolates; (**B**) CA: community-acquired clinical isolates; HA: hospital-acquired clinical isolates; HOSP: hospital environmental isolates; CT: cooling tower environmental isolates.

Reactivity against MAb 3/1 was assessed in 227 *L*. *pneumophila* sg 1 isolates, of which 49.78% were positive. Clinical isolates presented a greater proportion of MAb 3/1 positive isolates (79.0%) than environmental isolates (26.8%) (p < 0.0001) (Fig. 1A). The two clinical groups differed in the proportion of MAb 3/1 positive isolates (CA: 87.50% vs. HA: 47.83%, p < 0.0001) (Fig. 1B). Comparing the two major environmental groups, more MAb 3/1 positive isolates were observed in the cooling tower (CT) group than in the hospital (HOSP) group (27.40% vs. 5.26%, p = 0.03) (Fig. 1B).

Comparing clinical (CA and HA) with environmental (CT and HOSP) isolates, the CA isolates presented more MAb 3/1 positive isolates than both environmental groups (p < 0.005). In contrast, HA isolates presented a higher number of MAb 3/1 positives compared with HOSP isolates, (47.83% vs. 5.26%, p = 0.0055) (Fig. 1B) but not compared with CT isolates.

### MAb Dresden Panel subgrouping

The Dresden Panel divided all the *L*. *pneumophila* sg 1 isolates into 11 subgroups (Supplementary Data, Table S1). Clinical isolates were divided into only seven subgroups, with the Philadelphia (26.61%), Knoxville (19.27%) and OLDA (14.68%) and Benidorm (14.68%) subgroups being the most abundant. According to acquisition, the CA isolates were divided into six subgroups, in which Philadelphia (31.9%) and Knoxville (27.8%) were the most frequent, and HA isolates were divided into five subgroups, in which the most abundant were OLDA (24.24%) and *L*. *pneumophila* non-sg 1 (27.27%).

Environmental isolates were classified into the 11 subgroups of the Dresden Panel, with OLDA (33.1%) and *L*. *pneumophila* non-sg 1 (17.2%) being the most frequent. Of the two main environmental groups, CT isolates were classified into 10 subgroups, the most representative being OLDA (26.7%), Oxford (17.4%) and non-sg 1 (12.8%); HOSP isolates were classified into five Dresden subgroups with a predominance of OLDA (51.6%) and non-sg 1 (35.5%).

### SBT

The 266 isolates were divided into 90 different STs (Index of diversity (IOD): 0.940 CI: 0.925–0.955) (Supplementary Table S2).

**Figure 2:**
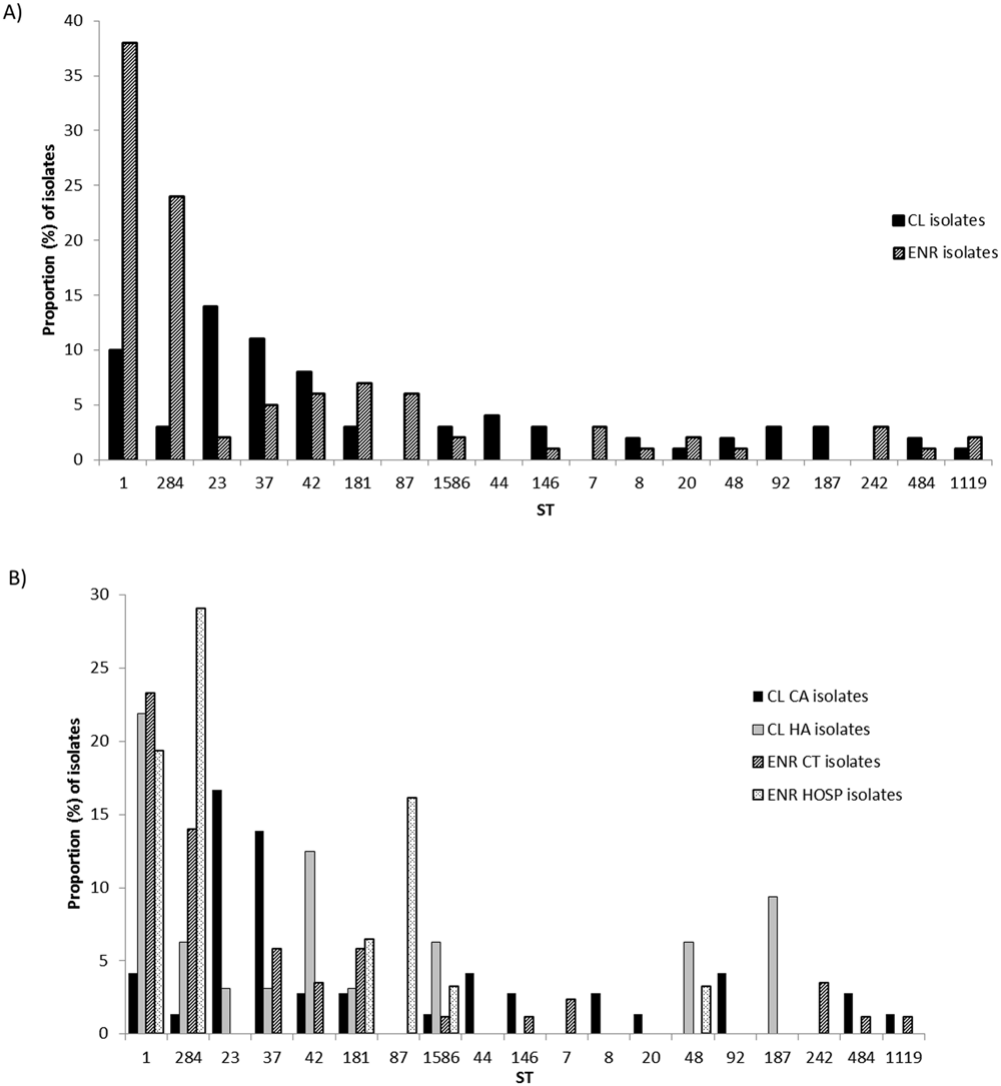
Distribution of the most frequent STs according to the origin. (**A**) CL: clinical isolates; ENR: environmental isolates; (**B**) CA: community-acquired clinical isolates; HA: hospital-acquired clinical isolates; HOSP: hospital environmental isolates; CT: cooling tower environmental isolates.

The 109 clinical isolates comprised 46 STs (IOD: 0.949; CI: 0.934–0.964), the most frequent being ST23 (n = 14, 12.84%), ST37 (n = 11; 10.09%), and ST1 (n = 10; 9.17%) (Fig. 2A). The CA group was represented by 38 STs (IOD: 0.936; CI: 0.907–0.965) among which ST23 (n = 12; 16.90%), and ST37 (n = 10; 14.08%) were the most abundant (Fig. 2B). The HA isolates presented only 17 different STs (IOD: 0.898; CI: 0.855–0.941), the most frequent being ST1 (n = 7; 21.88%), ST42 (n = 4; 12.50%), and ST187 (n = 3; 9.38%) (Fig. 2B). Only nine STs (ST1, ST23, ST37, ST42, ST181, ST193, ST284, ST436 and ST 1586) appeared in both groups.

The 157 environmental isolates were classified into 61 different STs (IOD: 0.908; CI: 0.877–0.938) and close to 40% were identified as ST1 (n = 38; 24.20%) or ST284 (n = 24; 15.29%) (Fig. 2A). CT isolates were divided into 39 different STs (IOD: 0.911; CI: 0.873–0.948) the most frequently identified being ST1 (n = 20; 23.26%) and ST284 (n = 12; 13.95%) (Fig. 2B). In contrast, HOSP isolates were divided into only 13 different STs (IOD: 0.839; CI: 0.767–0.911), with ST284 (n = 9; 29.03%), ST1 (n = 6; 19.35%) and ST87 (n = 5; 16.13%) predominating (Fig. 2B). Only five STs (ST1, ST181, ST284, ST728 and ST1586) were present in both CT and HOSP isolates.

### ST comparison between groups

The distribution of STs differed between groups. Twenty-nine out of 46 clinical STs appeared only in one group. Two of these 29 STs (ST193 and ST436) were found in both the CA and HA groups; 21 were found only in CA and six only in HA. Most of the environmental STs (44/61) did not appear in the clinical group. According to acquisition, only five STs (ST337, ST813, ST1836, ST1837 and ST2179) were exclusive to HOSP environments, 25 were exclusive to CT environments, one (ST728) was found in both, and the other 14 were found in other environments or in more than one environment.

Only four STs (ST1, ST181, ST284 and ST1586) were found in all the main groups (CA, HA, CT and HOSP).

Four out of 47 STs (8.5%) (ST1, ST181, ST284 and ST1586) appeared in the two most distant groups (CA and HOSP), according to the case-patient relationship and acquisition. However, when the closest clinical and environmental groups were compared (CA *vs*. CT and HA *vs*. HOSP), the number of common STs increased (CA *vs*. CT: 10/67 (14.9%); HA *vs*. HOSP: 6 out of 24 STs (25%) (6 STs: 1, 48, 181, 284, 1106 and 1586)).

### Phenons: combinations of SBT and MAb subgrouping

To obtain more discriminatory typing data, combinations of STs and MAb subgroups of the Dresden Panel were created, called phenons (ST-MAb subgroups) (Supplementary Data: Table S2). The 266 isolates were divided into 115 different phenons (IOD: 0.968; CI: 0.958–0.978): 53 different phenons in the clinical group (IOD: 0.969, CI: 0.957–0.981)) the most abundant being ST37-Philadelphia (n = 11, 10%) and ST23-Philadelphia (n = 10, 9.2%), and 75 in the environmental group (IOD: 0.955, CI: 0.937–0.974) the most abundant being ST1-OLDA (n = 26, 16.5%), ST284-OLDA (n = 16, 10.2%) and ST1-Oxford (n = 10, 6.4%).

The distribution of phenons varied between groups (Supplementary Data: Table S2). While CA clinical isolates were classified into 43 different phenons (CI: 0.965, IOD: 0.942–0.987), the most abundant being ST37-Philadelphia (n = 10, 13.9%) and ST23-Philadelphia (n = 8, 11.1%), HA isolates were divided into 17 phenons (IOD: 0.944, CI: 0.909–0.980), with ST1-OLDA (n = 5, 15.6%) and ST42-Benidorm (n = 4, 12.5%) as the most abundant.

CT environmental isolates were classified into 48 phenons (IOD: 0.969, CI: 0.953–0.986), the most representative being ST1-OLDA (n = 11, 12.8%) and ST1-Oxford (n = 7, 8.1%). The HOSP environmental isolates were separated into 13 different phenons (IOD: 0.877, CI: 0.805–0.950); ST284-OLDA (n = 9, 29%), ST1-OLDA (n = 5, 16%) and ST87-*L*. *pneumophila* non-sg 1 (n = 5, 16%) accounted for the majority of isolates.

### Phylogenetic analysis of ST

**Figure 3:**
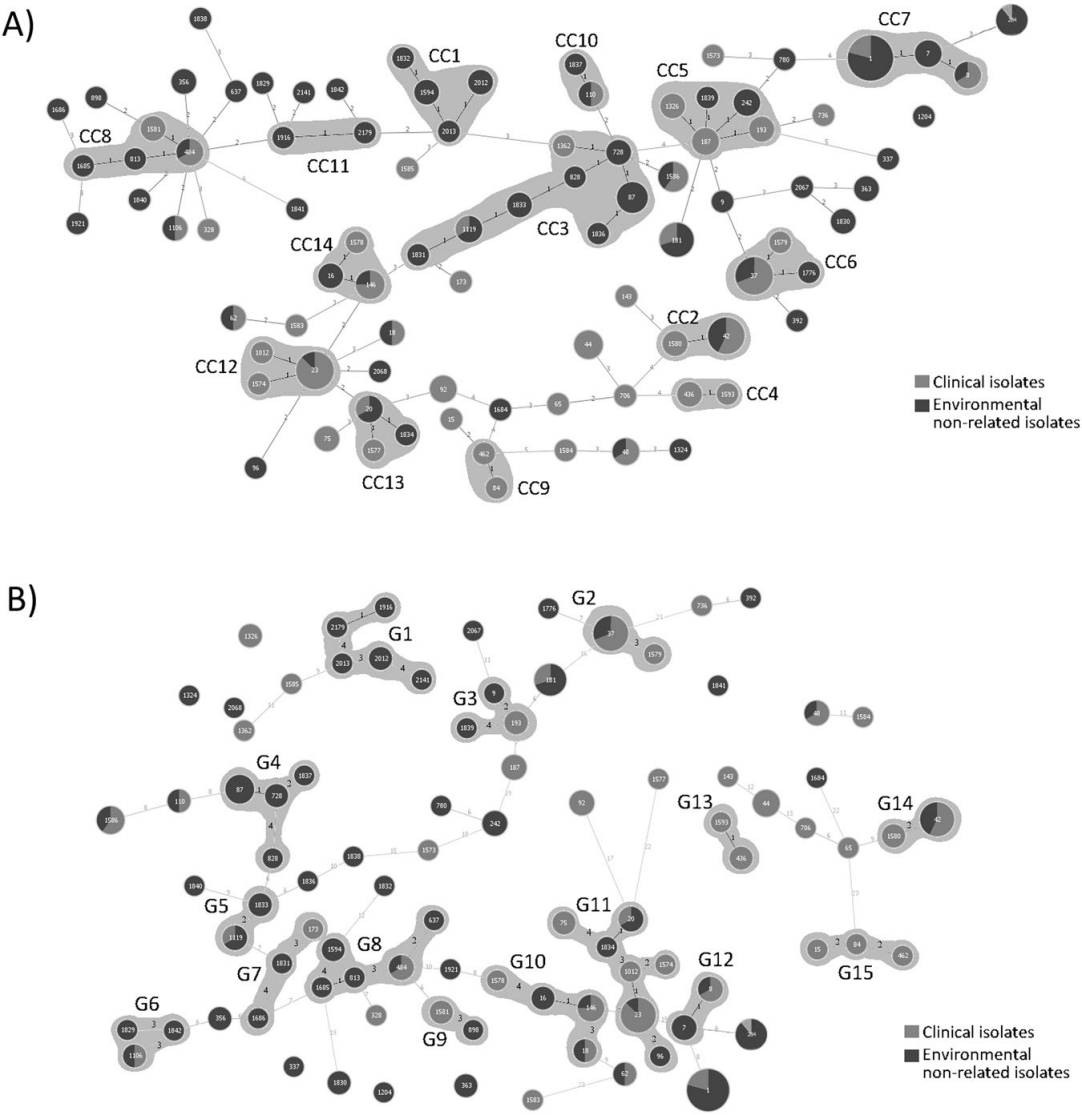
Minimum-spanning tree obtained with PhyloViz (**A**) based  on the SBT allelic profiles of *L*. *pneumophila* isolates from Catalonia divided into clinical and environmental isolates. Clonal Complexes (CC) of Single-Locus Variants are shaded. The size of each circle corresponds to the number of isolates within this particular type. Lines connecting STs show the number of gene variants.  (**B**) MST  based  on the SBT concatenated sequences. Groups (G) of < 5 bp differences among STs are shaded. The size of each circle corresponds to the number of isolates within this particular type. Lines connecting STs show the number of bp differences.

Minimum spanning trees (MST) using allelic profiles resulted in 14 clonal complexes (CC) and 44 singletons (STs which could not be assigned to any clonal complex) (Fig. 3A). The CCs with the most isolates were CC7 (which included ST1), CC6 (ST37), CC12 (ST23) and CC3. CC3 comprised the greatest number of STs, with a total of eight (ST728, ST87, ST1836, ST1362, ST828, ST1833, ST1119 and ST1831).

Phylogenetic analysis using concatenated sequences divided the isolates into 15 groups (G) and 39 individuals (STs that could not be assigned to any phylogenetic group) (Fig. 3B). The biggest group (G11) included seven STs. Seven out of the 39 individuals had more than 23 bp differences and the other 32 had between five and 23 bp differences with regard to each other. Interestingly, ST1 was found as an individual differing in 8 bp in the allele *neu* from ST7 (G12).

The cluster distribution of STs into the two analyses (allele (CC) or concatenated sequences (G)) presented differences. The STs that formed the different allelic CCs were divided into different concatenated Gs, and some singletons were linked to other STs which belonged to Gs. For example, G6 was made up of only singletons (ST1829, ST1842 and ST1106) with three bp differences between them. CC5 was completely deconstructed, forming G3 with only two STs (ST193 and ST1839) plus one singleton (ST9). The three other STs that constituted CC5 were found as individuals in the concatenated sequence analysis, two having between five and 23 bp differences (ST242 and ST187) and the other one having more than 24 bp differences (ST1326). Similarly, CC3 was divided into three different Gs (G4, G5 and G7) and two individuals had between five and 23 bp differences. G5 comprised two STs from CC3 (ST1883 and ST1119), while G4 consisted of three STs from CC3 (ST728, ST87 and ST828) and one ST from CC10 (ST1827). G7 comprised only one ST from CC3 (ST1831) and two singleton STs (ST173 and ST1686) which differed from ST1831 by only 3 and 4 bp, respectively.

## Discussion

The *L*. *pneumophila* population has been described in many countries^13–23^. Despite the high incidence of LD in Spain, the *L*. *pneumophila* population has been investigated in only two areas, the Comunitat Valenciana and Catalonia^9,13^: clinical and environmental *L*. *pneumophila* populations in the Comunitat Valenciana^13^, and only *L*. *pneumophila* clinical isolates in Catalonia^9^. In the present study, we compared clinical and environmental isolates of *L*. *pneumophila* recorded in Catalonia by SBT and MAb.

First, the isolates identified as *L*. *pneumophila* sg 1 were subgrouped by Dresden MAbs, including the virulence-associated MAb 3/1 (or MAb 2 in the Jolly panel^24^). The proportion of clinical MAb 3/1 positive isolates was higher than that of environmental isolates, as in other studies carried out in the United Kingdom, England-Wales and Canada (clinical: 79.6%, 96.6% and 63.6% *vs*. environmental: 12.8%, 8.3% and 17.1% respectively)^16,20,21^. Amemura-Maekawa *et al*. suggested that the MAb 3/1 epitope is easily lost or gained during adaptation to environments when there is no pressure to retain human pathogenicity^14^. Loss of the MAb 3/1 epitope may provide an advantage for fitness, while decreasing the potential to infect humans.

Furthermore, as regards acquisition, CA isolates that expressed the epitope Mab 3/1 were three times more frequent than HA isolates. This phenomenon was already observed in previous studies^20,25,26^, where it was suggested that the distribution of HA isolates is representative of environmental colonization and host susceptibility rather than of virulence traits^26^. Conversely, there was a greater proportion of MAb 3/1 positive isolates in the HA group than in the environmental HOSP group. This trend suggests that although patient susceptibility is important for HA cases, the ability of the isolates to cause infection may also be a risk factor.

For its part, the low number of MAb 3/1 negative isolates and the absence of *L*. *pneumophila* non sg 1 in the CA group could be due to an under-diagnosis of LD in the community, which may be favoured by the diagnostic tests currently used. Most of the reported cases of LD in Europe (88.6%)^4^ are diagnosed by urine antigen detection, but this diagnostic tool has been reported to be less sensitive to MAb 3/1 negative isolates and non-specific for *L*. *pneumophila* non sg 1^27,28^. Furthermore, HA cases present an advantage in that the sample is collected before the treatment of LD and after the appearance of the first symptoms, which helps to obtain isolates from respiratory samples.

The high SBT diversity observed in the isolates analysed was comparable to the clinical and environmental diversity found in other larger countries (UK, US and Canada)^16,20,23^. Given that the clinical isolates came from the environment, we expected them to be less diverse than the environmental isolates, but in fact, corroborating previous findings from the USA and Canada study^15,20^, they had greater diversity. The diversity of environmental SBT in Japan and the US showed that artificial environments present a trend towards a lower IOD^14,15^. The environmental isolates from Catalonia had a high diversity, although all the isolates came from artificial environments. The factors affecting the degree of genetic diversity in certain areas are unknown. Possible explanations include variations in the quality of the water or in the disinfection measures applied due to the differences in environmental regulations. Indeed, even according to origin, the diversity of the environmental isolates in this study continued to be higher than in other countries. The environmental HOSP group comprised a less diversified population and was divided into only 13 STs, with ST284 predominating (29%), followed by ST1 (19%). The diversity of this group of isolates was similar to that of the artificial environmental isolates in Japan and China^14,29^. The diversity of HA was similar to that of the environmental isolates and slightly higher than that of the HOSP isolates. Furthermore, the distribution of the HA isolates mirrored that of the environmental and HOSP isolates, supporting the notion that HA isolate distribution may be representative of environmental colonisation in which the virulence traits of the strains are less relevant than the intrinsic risk factors of inpatients. Despite these similarities, the proportion of MAb 3/1 positive isolates was higher in the HA isolates; this might be explained by the hypothesis of Amemura-Maekawa *et al*.^14^, which posits that the MAb 3/1 epitope is gained and lost according to the environmental pressure (that is, the need to retain human pathogenicity).

A total of 90 STs were identified, and only 17 were shared between clinical and environmental isolates. This difference in the distribution of STs has been reported in previous studies^16,20^. ST1 was the most frequent, accounting for almost 18% of the isolates. This ST^14,20,25,30–32^ is found worldwide, and is the most abundant in the EWGLI database (13%). In our study, clinical isolate ST1 was found in 9.17%, and tended to be more frequent in the HA isolates. In our study and in the 2014 US study^15^, ST1 was found more frequently in environmental than in clinical isolates. The reason for the low concordance between environmental and clinical frequency in our area is unknown, but there are several possible explanations. The first is the unavailability of ST1 clinical isolates in our database due to difficulties in culturing respiratory samples. Second, ST1 may be well adapted to the environment, with a low ability to infect, and may be more likely to trigger LD in hosts with a high level of risk factors such as HA cases.

In the present study, ST23 and ST37 were the most frequently found in the clinical group. In France, ST23^22^ was the most frequently found in the clinical group together with ST1 and ST47, and, as in our study, it was rarely found in the environmental group. In the MST analysis ST23 was located in CC12 with ST1012 and ST1574, both of which were only found as clinical STs, with one and three bp differences in the ST23-*mompS* gene respectively. As other authors have suggested with other sets of STs^15,33^, this ST complex probably has a high capacity for infection, albeit with a reduced persistence in the environment. The MST analysis by concatenated sequences showed a grouping of these three STs in the G11 with four other STs: two STs were singletons in the CC analysis (ST75 only in the clinical group, and ST96 only in the environmental group) and two STs belonged to the CC13 (ST1874 only in the environmental group, ST20 in both clinical and environmental groups).

All the ST37 isolates in this study were characterised as MAb 3/1 positive, subgroup Philadelphia. This ST is related to ST36 and ST35, which were reported to be the cause of a large number of cases of LD in Europe and the US^15^. Harrison *et al*. suggested that these STs belonged to a ST group with an increased capacity to trigger LD^33^. ST37 was responsible for a large outbreak in Catalonia in 2002^34^, and the largest outbreak described in Murcia, Spain in 2000^35^. In the MST analysis ST37 was located in CC6 together with ST1579, which was only found in clinical isolates, and ST1776, which was only found in environmental isolates. ST37 had seven bp differences compared to ST1776 in the *neuA* gene and three bp differences compared to ST1579 in the *asd* gene. Therefore, in the concatenated analysis ST37 was only linked to ST1579, the clinical ST; together they formed the G2. MST analysis by concatenated sequences may be a useful tool for extrapolating information about the variation in genes (the different alleles of each gene) and their role implication.

The most frequent environmental STs were ST1, ST284 and ST181, which accounted for 54.3% of the environmental *L*. *pneumophila* sg 1 isolates. The most representative ST from the *L*. *pneumophila* non sg 1 isolates was ST87 (22.2%). Together with ST42, these three isolates (ST1, ST284 and ST181) were the most common in HA isolates (43%), but were only found in 11% of CA isolates. Moreover, these three STs together with ST87 made up 70% of the HOSP isolates. This relative homology between HA and HOSP indicates that the relationship between the presence of the isolates and patient susceptibility is a possible cause of LD development.

Compared to all the other isolates, the CA group was the most differentiated, although the MST analyses did not show a cluster group that differed from the others. Interestingly, the CA isolates were similar to those from the regions studied in the US and Europe^16,20,23^. Comparing the presence of all the environmental STs (except ST1) in Catalonia with other regions in the EWGLI database^36^, the environmental isolates were found to be more heterogeneous than clinical STs. These results could be due, in part, to the low number of environmental studies and the low rate of ST notification to the EWGLI database.

The classification of isolates by phenons increased the diversity, but few phenons comprised the majority of clinical or environmental isolates. These findings corroborate those of a UK study^16^ where 45% of clinical isolates were divided into four STs and 31% of environmental isolates were divided into three STs.

A major limitation of this study was that the isolates included were from epidemiological studies performed in our laboratory. This may have introduced a bias because current diagnostic methods under-diagnose LD cases caused by *L*. *pneumophila* non-sg 1 and *L*. *pneumophila* sg 1 MAb 3/1 negative isolates; what is more, since the environmental isolates came from case/outbreak investigations (i.e., they were not random either in terms of location or of time), we cannot extrapolate our results to routine environmental samples (i.e., samples from quality and prevention programs) and they may not provide an accurate reflection of the population structure of the area studied. This bias might be increased further by the fact that the majority of clinical isolates were *L*. *pneumophila* sg 1, and in the selection of the environmental isolates for the typing process most of the non sg 1 isolates were discarded. Therefore, although some non sg 1 were characterised, their results are not representative of this population; the possibility of other subtypes coexisting in the environment in addition to the ones included cannot be ruled out. Another limitation may be the variations in the surveillance systems and methods used to detect *Legionella* from hospital to hospital, which means that we cannot be sure that all LD cases were tested for *Legionella* during the study period. Likewise, the low productivity of sputum cultures made it difficult to obtain more clinical isolates for evaluation. All these limitations were increased by subdividing the isolates into smaller groups; although a trend was found in each subgroup, the differences observed may not represent a true reflection of the population structure.

In addition, although SBT is considered the gold standard, this method is based on the characterisation of only seven genes. The analysis of 2.5 kb out of 3.5 Mb of the genome^37–39^ must be interpreted with caution, because it may not present a true reflection of the population structure. For example, in previous studies when unrelated ST1 isolates were typed by other molecular methods, the isolates were divided into different subtypes^40–42^.

This study presents a description and comparison of Catalan *L*. *pneumophila* environmental and clinical isolates. With regard to MAb subgrouping and ST typing, the clinical CA isolates were the group that differed the most from the others, with a higher proportion of MAb 3/1 positive isolates and fewer shared STs. Their capacity to infect and their adaptability to the environment may be due to the acquisition of some virulent or adaptability traits by horizontal gene transfer from other isolates coexisting in the same environmental source^43,44^. Likewise, some isolates could be better adapted to the environment or more prone to infect humans. For its part, the high similarity between HOSP and HA isolates may be more closely related to the abundance of these isolates in the environment and to patient susceptibility than to the virulence capacity itself.

### Future perspectives

Although this study describes the distribution and classification of environmental and clinical STs in Catalonia, further studies are needed using whole genome sequencing (WGS) as a tool to classify the isolates and to identify the factors/genes that enhance the ability to infect humans.

The major drawback of WGS is the use of different reference genomes and bioinformatics pipelines and the lack of consensus regarding the establishment of a minimum number of different pb to determine whether two isolates are identical. As shown in the two phylogenetic approach analyses in the present study, the classification of the isolates varies according to whether allelic (CC) or concatenated sequences (G) are used. Nor is there any consensus regarding the limit of sequence variances needed to accurately interpret the large amount of data obtained using this method. However, the high level of discrimination offered by WGS^45–47^ makes it a good tool for epidemiological studies. To date, most WGS studies have used a single nucleotide polymorphism (SNP)-based approach and have found the related environmental and clinical isolates to be <20 SNPs apart^48,49^. Currently an extended core genome MLST (cgMLST) scheme with approximately 50–100 core genes^47^ is being designed and tested for *L*. *pneumophila*. This new approach has advantages over other analyses of WGS data and may establish itself as the new gold standard for population structure analysis.

## Methods

### Bacterial isolates

A total of 528 isolates of *L*. *pneumophila* from Catalonia were collected for the study. These isolates came from epidemiological studies of LD conducted between 1989 and 2016. Isolates were maintained in glycerol-Brain Heart Infusion (BHI) (Oxoid) at −80 °C. They were seeded and grown on BCYE agar plates incubated at 37 °C during 72 hours, and were evaluated by MAb typing and SBT.

The study was conducted in accordance with the Declaration of Helsinki and Spanish legislation concerning clinical research. The protocols were approved by the local Human Research Ethical Committee (CEIC-HUGTP, Badalona). Since 2001, our laboratory has been the Catalan Reference Laboratory for *Legionella* typing in LD outbreak/cluster investigations. Because the health authorities referred anonymised clinical isolates for molecular typing to our laboratory, a waiver for informed consent was obtained from the local Human Research Ethical Committee in accordance with Spanish law.

#### Clinical isolates

A total of 159 clinical isolates were collected and characterised (1 isolate/patient). Among the related isolates from the same outbreak (from patients with a probable or confirmed common source of infection), the first isolate from each outbreak was included in the analysis as a representative of the case/outbreak group, and 109 clinical isolates were fully analysed. Clinical isolates were divided according to the source of acquisition into: Hospital-acquired (HA = 32), Community-acquired (CA = 72) and unknown origin (UO = 5) (epidemiological data not available). In a previous study^9^ we characterised 95 out of these 109 isolates by SBT and MAb subgrouping.

#### Environmental isolates

We included 369 environmental *L*. *pneumophila* isolates recovered and isolated during epidemiological studies of LD cases between 1989 and 2016.

Environmental isolates related to LD cases were excluded from the analyses, and the isolates which presented the same ST and MAb from the same point analysed were eliminated in order to reduce the redundancy of the results. One hundred and fifty-seven environmental isolates were fully analysed. These isolates were classified according to the origin of the sample: Cooling Tower (CT, n = 86), Hotel (n = 4), Hospital (HOSP, n = 31), Water Distribution System (WDS, n = 12), Other (spas, sprinklers, tanks and pools; n = 8) and Unknown (data not available, n = 16).

#### L. pneumophila identification, MAb 3/1 reactivity and MAb Dresden Panel subgrouping

*L*. *pneumophila* isolates were identified by the Monofluo IFA test kit (Genetic Systems Corp., Redmond, WA, USA) and were differentiated as serogroup 1 (sg 1) or non-serogroup 1 by immunoagglutination serotyping (Oxoid *Legionella* Latex; Germany).

MAbs from the Dresden Panel were used to determine the serogroup of *L*. *pneumophila* and the phenotypic subgroup of *L*. *pneumophila* serogroup 1. The determination of the serogroup is based on the reaction against a specific antibody by indirect immunofluorescence for each serogroup, and the phenotypic subgroup of *L*. *pneumophila* sg 1 is based on the reaction against seven MAbs by an indirect immunofluorescence assay, and by dividing sg 1 into nine different phenotypic subgroups according to a flow chart^26^.

### SBT

Genomic DNA was extracted using the Chelex™ extraction technique (Bio-Rad Laboratories, CA, USA). The seven target genes (*flaA*, *pilE*, *asd*, *mip*, *mompS*, *proA*, and *neuA*) were amplified using the primers and amplification protocol provided by the EWGLI (4.2). DNA sequencing was performed on a 3130xl (3100) ABI Prism genetic analyser (Applied Biosystems) at the Genomics Core Facility at the Germans Trias i Pujol Research Institute. Sequences were analysed using Sequence Scanner software v1.0 (Applied Biosystems). Then, the allele number was assigned using the SBT-EWGLI Quality tool, and the ST profiles were defined with the SBT-EWGLI database.

### Data interpretation

The diversity index (IOD) for each variable was calculated using the Hunter Gaston index with the VDICE online tool (http://www.hpa-bioinformatics.org.uk/cgi-bin/DICI/DICI.pl).

Minimum spanning trees (MST) were created by goeBURST implemented in PHYLOViz^50^. STs were represented by circles; the size of a circle indicates the number of isolates of this particular ST. Two approaches were performed: conventional MST using allelic profiles, and concatenated sequences of the alleles. The clonal complexes (CC) were calculated with the allelic profiles and were defined using the criteria of single locus variant. STs that cannot be assigned to any clonal complex are called singletons.

The Groups (G) in the MST using the concatenated sequences were restricted to differences of 4 bp or less between STs. After several analyses (data not shown), we chose a cut-off of 4 bp which resulted in a similar number of Groups (15) to CCs (14). STs that cannot be assigned to any phylogenetic Group are called individuals.

### Statistical analysis

Statistical analyses were carried out using SPSS statistical package program software (version 19 SPPS, Chicago IL, USA). The results for categorical variables were expressed as absolute and relative frequencies and medians and interquartiles. The Chi Square test was used to compare the proportional distributions among groups. The statistical tests used in the study were two-sided, and a p value of 0.05 or less was considered statistically significant.

## Electronic supplementary material

Table S1

Table S2

## References

[1] Sopena N (1999). Prospective study of community-acquired pneumonia of bacterial etiology in adults. Eur. J. Clin. Microbiol. Infect. Dis. Off. Publ. Eur. Soc. Clin. Microbiol..

[2] Benin AL, Benson RF, Besser RE (2002). Trends in Legionnaires Disease, 1980–1998: Declining Mortality and New Patterns of Diagnosis. Clin. Infect. Dis..

[3] Yu VL (2002). Distribution of *Legionella* Species and Serogroups Isolated by Culture in Patients with Sporadic Community‐Acquired Legionellosis: An International Collaborative Survey. J. Infect. Dis..

[4] European Centre for Disease Prevention and Control. Legionnaires disease in Europe, 2014. *ECDC*, Stockholm, Sweden (2017).

[5] Sala MR (2007). Community outbreak of Legionnaires disease in Vic-Gurb, Spain in October and November 2005. Euro Surveill..

[6] de Olalla PG (2008). [Community outbreak of pneumonia due to *Legionella pneumophila*: importance of monitoring hospital cooling towers]. Enfermedades Infecc. Microbiol. Clínica.

[7] Barrabeig I (2010). Outbreak of Legionnaires’ disease associated with a supermarket mist machine. Epidemiol. Infect..

[8] Rota, M. *et al*. Cluster of travel-associated Legionnaires disease in Lazise, Italy, July to August 2011. *Euro Surveill*. **16** (2011). 10.2807/ese.16.40.19982-en 21996379

[9] Garcia-Nuñez M (2016). Characterization of unrelated clinical *Legionella pneumophila* isolates in Catalonia by monoclonal subgrouping and sequence-based typing. Future Microbiol..

[10] Gaia V (2005). Consensus sequence-based scheme for epidemiological typing of clinical and environmental isolates of *Legionella pneumophila*. J. Clin. Microbiol..

[11] Gaia V, Fry NK, Harrison TG, Peduzzi R (2003). Sequence-based typing of *Legionella pneumophila* serogroup 1 offers the potential for true portability in legionellosis outbreak investigation. J. Clin. Microbiol..

[12] Ratzow S, Gaia V, Helbig JH, Fry NK, Lück PC (2007). Addition of *neuA*, the gene encoding N-acylneuraminate cytidylyl transferase, increases the discriminatory ability of the consensus sequence-based scheme for typing *Legionella pneumophila* serogroup 1 strains. J. Clin. Microbiol..

[13] Sánchez-Busó L (2015). Geographical and Temporal Structures of *Legionella pneumophila* Sequence Types in Comunitat Valenciana (Spain), 1998 to 2013. Appl. Environ. Microbiol..

[14] Amemura-Maekawa J (2012). Distribution of monoclonal antibody subgroups and sequence-based types among *Legionella pneumophila* serogroup 1 isolates derived from cooling tower water, bathwater, and soil in Japan. Appl. Environ. Microbiol..

[15] Kozak-Muiznieks NA (2014). Prevalence of sequence types among clinical and environmental isolates of *Legionella pneumophila* serogroup 1 in the United States from 1982 to 2012. J. Clin. Microbiol..

[16] Harrison TG, Afshar B, Doshi N, Fry NK, Lee JV (2009). Distribution of *Legionella pneumophila* serogroups, monoclonal antibody subgroups and DNA sequence types in recent clinical and environmental isolates from England and Wales (2000-2008). Eur. J. Clin. Microbiol. Infect. Dis..

[17] Chasqueira, M. J., Rodrigues, L., Nascimento, M. & Marques, T. Sequence-based and monoclonal antibody typing of *Legionella pneumophila* isolated from patients in Portugal during 1987-2008. *Euro Surveill*. **14** (2009). 10.2807/ese.14.28.19271-en 19607780

[18] Bianchi A, Pregliasco FE, Consonni M, Tesauro M (2016). Genotypic diversity of *Legionella pneumophila* in environmental and clinical strains assessed by Sequence-Based Typing, in association with retrospective clinical surveillance in Northern Italy. Ann. Agric. Environ. Med. AAEM.

[19] Lim YH (2011). Environmental surveillance and molecular characterization of *Legionella* in tropical Singapore. Trop. Biomed..

[20] Reimer AR, Au S, Schindle S, Bernard KA (2010). L*egionella pneumophila* monoclonal antibody subgroups and DNA sequence types isolated in Canada between 1981 and 2009: Laboratory Component of National Surveillance. Eur. J. Clin. Microbiol. Infect. Dis..

[21] Harrison TG, Doshi N, Fry NK, Joseph CA (2007). Comparison of clinical and environmental isolates of *Legionella pneumophila* obtained in the UK over 19 years. Clin. Microbiol. Infect..

[22] Cassier P (2015). Epidemiologic characteristics associated with ST23 clones compared to ST1 and ST47 clones of Legionnaires disease cases in France. New Microbes New Infect..

[23] Kozak NA (2009). Distribution of lag-1 alleles and sequence-based types among *Legionella pneumophila* serogroup 1 clinical and environmental isolates in the United States. J. Clin. Microbiol..

[24] Joly JR (1986). Development of a standardized subgrouping scheme for *Legionella pneumophila* serogroup 1 using monoclonal antibodies. J. Clin. Microbiol..

[25] Borchardt J, Helbig JH, Lück PC (2008). Occurrence and distribution of sequence types among *Legionella pneumophila* strains isolated from patients in Germany: common features and differences to other regions of the world. Eur. J. Clin. Microbiol. Infect. Dis..

[26] Helbig JH (2002). Pan-European study on culture-proven Legionnaires’ disease: distribution of *Legionella pneumophila* serogroups and monoclonal subgroups. Eur. J. Clin. Microbiol. Infect. Dis..

[27] Helbig JH, Uldum SA, Lück PC, Harrison TG (2001). Detection of *Legionella pneumophila* antigen in urine samples by the BinaxNOW immunochromatographic assay and comparison with both Binax *Legionella* Urinary Enzyme Immunoassay (EIA) and Biotest *Legionella* Urin Antigen EIA. J. Med. Microbiol..

[28] Jørgensen CS (2015). Evaluation of a new lateral flow test for detection of *Streptococcus pneumoniae* and *Legionella pneumophila* urinary antigen. J. Microbiol. Methods.

[29] Guo J (2015). Sequence types diversity of L*egionella pneumophila* isolates from environmental water sources in Guangzhou and Jiangmen, China. Infect. Genet. Evol..

[30] Amemura-Maekawa J (2010). Characterization of *Legionella pneumophila* isolates from patients in Japan according to serogroups, monoclonal antibody subgroups and sequence types. J. Med. Microbiol..

[31] Lee HK, Shim JI, Kim HE, Yu JY, Kang YH (2010). Distribution of *Legionella* species from environmental water sources of public facilities and genetic diversity of *L*. *pneumophila* serogroup 1 in South Korea. Appl. Environ. Microbiol..

[32] Tijet N (2010). New endemic *Legionella pneumophila* serogroup I clones, Ontario, Canada. Emerg. Infect. Dis..

[33] Harrison TG *et al*. Typing of *Legionella pneumophila* and its role in elucidating the epidemiology of Legionnaire’s disease. in *Legionella: state of the art 30 years after its recognition* 94–99 (ASM Press) (2006).

[34] Garcia-Nuñez M (2013). Comparative molecular and antibody typing during the investigation of an outbreak of Legionnaires’ disease. J. Infect. Chemother..

[35] García-Fulgueiras A (2003). Legionnaires’ disease outbreak in Murcia, Spain. Emerg. Infect. Dis..

[36] EWGLI | The European Working Group for *Legionella* Infections | Home. Available at: http://www.ewgli.org/.

[37] Sánchez-Busó L, Coscollá M, Pinto-Carbó M, Catalán V, González-Candelas F (2013). Genetic Characterization of *Legionella pneumophila* Isolated from a Common Watershed in Comunidad Valenciana, Spain. PloS One.

[38] Cazalet C (2004). Evidence in the *Legionella pneumophila* genome for exploitation of host cell functions and high genome plasticity. Nat. Genet..

[39] Chien M (2004). The genomic sequence of the accidental pathogen *Legionella pneumophila*. Science.

[40] Quero S (2016). Discriminatory usefulness of pulsed-field gel electrophoresis and sequence-based typing in *Legionella* outbreaks. Future Microbiol..

[41] Ginevra C (2012). *Legionella pneumophila* sequence type 1/Paris pulsotype subtyping by spoligotyping. J. Clin. Microbiol..

[42] David S (2017). Seeding and Establishment of *Legionella pneumophila* in Hospitals: Implications for Genomic Investigations of Nosocomial Legionnaires’ Disease. Clin. Infect. Dis..

[43] Underwood AP, Jones G, Mentasti M, Fry NK, Harrison TG (2013). Comparison of the *Legionella pneumophila* population structure as determined by sequence-based typing and whole genome sequencing. BMC Microbiol..

[44] Gomez-Valero L (2011). Extensive recombination events and horizontal gene transfer shaped the *Legionella pneumophila* genomes. BMC Genomics.

[45] Qin T (2016). Population structure and minimum core genome typing of *Legionella pneumophila*. Sci. Rep..

[46] Bosch T (2015). Whole-Genome Mapping as a Novel High-Resolution Typing Tool for *Legionella pneumophila*. J. Clin. Microbiol..

[47] David S (2016). Evaluation of an Optimal Epidemiological Typing Scheme for *Legionella pneumophila* with Whole-Genome Sequence Data Using Validation Guidelines. J. Clin. Microbiol..

[48] Graham RMA, Doyle CJ, Jennison AV (2014). Real-time investigation of a L*egionella pneumophila* outbreak using whole genome sequencing. Epidemiol. Infect..

[49] Reuter, S. *et al*. A pilot study of rapid whole-genome sequencing for the investigation of a *Legionella* outbreak. *BMJ Open* **3** (2013). 10.1136/bmjopen-2012-002175 PMC3553392 23306006

[50] Francisco AP, Bugalho M, Ramirez M, Carriço JA (2009). Global optimal eBURST analysis of multilocus typing data using a graphic matroid approach. BMC Bioinformatics.

