# Supplementary material for: Population structure of Environmental and Clinical Legionella pneumophila isolates in Catalonia
Source: Sci Rep. 2018 Apr 19;8:6241. doi: 10.1038/s41598-018-24708-1 (PMC5908911; doi:10.1038/s41598-018-24708-1)
Supplement: Supplementary file 1 — Table S1 [file 41598_2018_24708_MOESM1_ESM.doc]

**Title**: Population structure of Environmental and Clinical *Legionella pneumophila* isolates in Catalonia.

Authors: Sara Quero, Noemí Párraga-Niño, Irene Barrabeig, Maria Rosa Sala, M Luisa Pedro-Botet, Eduard Monsó, Mireia Jané, Miquel Sabrià, Marian Garcia-Núñez

Table S1. Dresden subgrouping according to the clinical (CL) and environmental (ENV) groups. WDS water distribution systems. The total number of isolates in each group is shown in parenthesis.

|  | **Knoxville** | **Philadelphia** | **Benidorm** | **France/**  **Allentown** | **OLDA** | **Oxford** | **Oxford/**  **OLDA** | **Bellingham** | **Heysham** | **Camperdown** | **Lp non-sg1** | **Indetermined** |
| --- | --- | --- | --- | --- | --- | --- | --- | --- | --- | --- | --- | --- |
| **CL** | **19.27 % (21)** | **26.61 %**  **(29)** | **14.68 % (16)** | **11.93 % (13)** | **14.68 % (16)** |  |  | **4.59 %**  **(5)** |  |  | **8.26 %**  **(9)** |  |
| CL CA | 27.78 % (20) | 31.94 %  (23) | 11.11 % (8) | 16.67 % (12) | 11.11 % (8) |  |  | 1.39 %  (1) |  |  |  |  |
| CL HA |  | 15.15 %  (5) | 18.18 % (6) |  | 24.24 % (8) |  |  | 12.12 % (4) |  |  | 27.27 % (9) |  |
| CL Unknown | 20.00 % (1) | 20.00 %  (1) | 40.00 % (2) | 20.00 % (1) |  |  |  |  |  |  |  |  |
|  |  |  |  |  |  |  |  |  |  |  |  |  |
| **ENV** | **7.01 % (11)** | **3.82 %**  **(6)** | **10.19 % (16)** | **0.64 %**  **(1)** | **33.12 % (52)** | **12.74 % (20)** | **0.64 % (1)** | **8.28 % (13)** | **3.18 %**  **(5)** | **1.27 %**  **(2)** | **17.20 % (27)** | **1.91 % (3)** |
| ENV CT | 6.98 %  (6) | 4.65 %  (4) | 11.63 % (10) |  | 26.74 % (23) | 17.44 % (15) | 1.16 % (1) | 10.47 % (9) | 3.49 %  (3) | 2.33 %  (2) | 12.79 % (11) | 2.33 % (2) |
| ENV HOSP |  |  | 3.23 % (1) |  | 51.61 % (16) | 3.23 % (1) |  | 3.23 %  (1) |  |  | 35.48 % (11) | 3.23 % (1) |
| ENV WDS | 16.67 % (2) |  | 25.00 % (3) | 8.33 %  (1) | 33.33 % (4) |  |  |  |  |  | 16.67 % (2) |  |
| ENV Hotel | 25.00 % (1) | 25.00 %  (1) | 50.00 % (2) |  |  |  |  |  |  |  |  |  |
| ENV Others | 12.50 % (1) | 12.50 %  (1) |  |  | 37.50 % (3) |  |  |  | 12.50 % (1) |  | 25.00 % (2) |  |
| ENV Unknown | 6.25 %  (1) |  |  |  | 35.50 % (6) | 25.00 % (4) |  | 18.75 % (3) | 6.25 %  (1) |  | 6.25 %  (1) |  |
